# Management of Nonpuerperal Uterine Inversion Using a Combined Vaginal and Abdominal Approach

**DOI:** 10.1155/2020/8827207

**Published:** 2020-12-28

**Authors:** Ferid A. Abubeker, Mulugeta Misgina, Ahmed Ebabu, Eyerusalem Fekade, Biruck Gashawbeza

**Affiliations:** ^1^Department of Obstetrics and Gynecology, Saint Paul's Hospital Millennium Medical College, P.O. Box 29497, Addis Ababa, Ethiopia; ^2^Department of Pathology, Saint Paul's Hospital Millennium Medical College, Addis Ababa, Ethiopia

## Abstract

**Introduction:**

Nonpuerperal uterine inversion is an extremely rare clinical condition. As such, some cases will have to be managed without prior experience. Clinicians must have a high index of suspicion to make the diagnosis and a clear understanding of the principles of recommended surgical techniques. Here, we report a case of nonpuerperal uterine inversion managed using a combined vaginal and abdominal approach. *Case Presentation*. A 70-year-old postmenopausal woman presented with profuse vaginal bleeding and protruding mass per vagina. Examination showed a solitary globular mass attached to an inverted uterus. A clinical diagnosis of nonpuerperal uterine inversion was made. A vaginal approach was used to first remove the mass followed by an abdominal approach to reposition the uterus using the *Haultain procedure*. Subsequently, total abdominal hysterectomy with bilateral salpingo-oophorectomy was done without complication. Histologic examination showed myoma with adenomyosis.

**Conclusion:**

Advanced imaging techniques such as 3D power Doppler and MRI have signature signs to confirm the clinical diagnosis of uterine inversion. Short of these diagnostic modalities, however, carefully conducted clinical examination including examination under anesthesia, and pelvic ultrasonography can be valuable tools to reach at a diagnosis. A combined vaginal and abdominal surgical approach can facilitate repositioning and/or hysterectomy when there is a large protruding vaginal mass.

## 1. Introduction

Uterine inversion is a clinical condition characterized by invagination of the uterine fundus into the endometrial cavity to or through the cervix. Puerperal uterine inversion is a rare complication of delivery with an incidence of 1 in 3,500 deliveries [1]. Nonpuerperal uterine inversion is even more rare and is almost always associated with uterine tumors. Submucosal leiomyomas are considered the most frequent cause though malignant conditions are occasionally encountered [2].

Several surgical techniques have been described to manage uterine inversion [3]. However, some cases will have to be managed without prior experience as nonpuerperal uterine inversion is a rare clinical encounter. This requires a high index of suspicion to make the diagnosis and a clear understanding of the principles of recommended surgical techniques. Here, we report a case of nonpuerperal uterine inversion managed using a combined vaginal and abdominal approach.

## 2. Case Presentation

A 70-year-old postmenopausal woman was referred to our gynecologic emergency unit with an impression of endometrial cancer after she presented with profuse vaginal bleeding of 3 hours associated with lower abdominal pain and protruding mass per vagina of 20 hours. She noticed the mass after experiencing urinary retention and was straining to void her bladder.

The history revealed intermittent vaginal bleeding and progressively worsening voiding difficulties for the past 3 months. Four weeks earlier, she visited a local health facility where transabdominal ultrasonography was done and showed intrauterine echocomplex mass consistent with leiomyoma. A cervical punch biopsy was found to be negative for malignancy. She had two uncomplicated term vaginal deliveries. She is a known diabetic patient taking insulin for the past 8 years.

**Figure 1:**
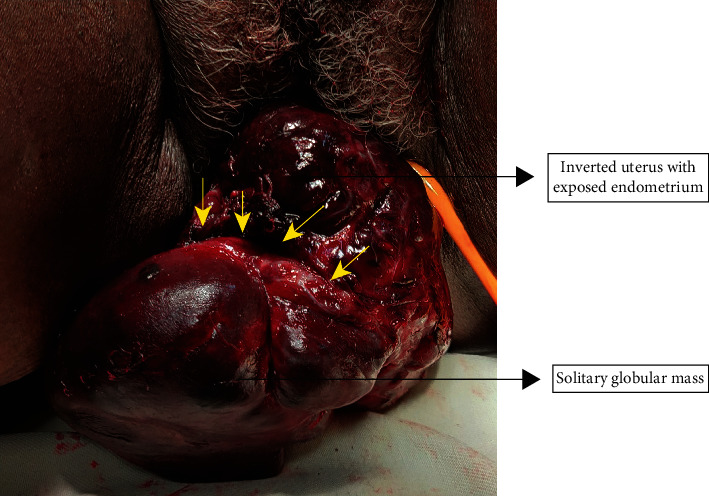
Inverted uterus with a solitary mass attached to an exposed endometrial surface (yellow arrows).

On examination, she had tachycardia (118 bpm) and blood pressure of 90/60 mmHg. The patient looked pale. Gynecologic examination revealed an irregular beefy red mass measuring about 15 × 10 cm protruding through the vaginal introitus. The mass was noted to have two distinct parts with a clear demarcation (Figure 1). The lower part was a solitary globular mass with a firm consistency and smooth surface. The upper part had an irregular sloughing surface with areas of necrosis and contact bleeding. The cervix could neither be seen nor palpated. It was difficult to appreciate the uterus on bimanual examination due to tenderness.

The patient had a hemoglobin of 10.3 g/dl. Renal function test, blood sugar, and ketone levels were within the normal range.

Transabdominal ultrasonography could not identify the uterus in the pelvic cavity. The patient was consented for surgery with a tentative diagnosis of nonpuerperal uterine inversion due to leiomyoma. However, due to the age of the patient and the features of the mass, the possibility of malignancy could not be excluded. A combined vaginal and abdominal approach was planned.

**Figure 2:**
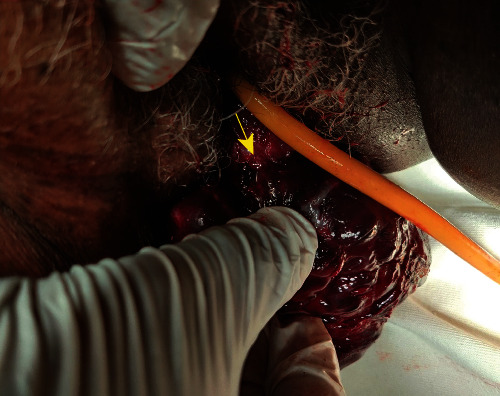
Tight cervical ring (yellow arrow) behind the mass.

In the operating room, examination under anesthesia was done and a hard and tight cervical ring was identified behind the mass (Figure 2).

During laparotomy, the diagnosis of uterine inversion was confirmed. The uterus was absent, and the bilateral infundibulopelvic, uteroovarian, and round ligaments and fallopian tubes were pulled medially into a central constriction ring. Only the fimbrial end of the fallopian tubes and the ovaries were visible at the edge of the constriction ring. The liver, omentum, and bowel loops appeared normal.

**Figure 3:**
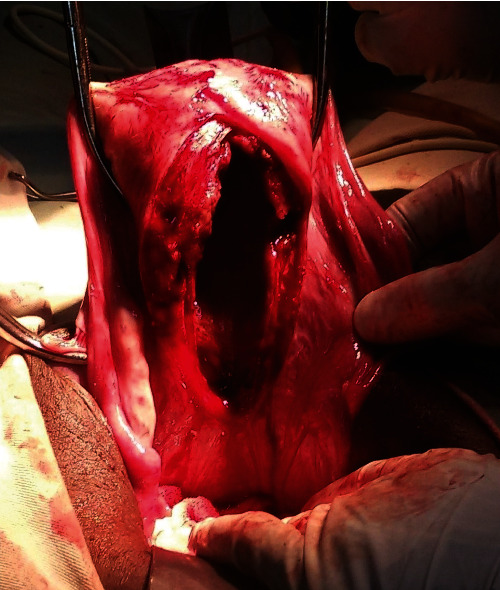
Abdominal view of the posterior uterine wall after repositioning using the *Haultain* procedure.

The surgeon first removed the tumor to reduce the volume of the mass protruding from the vagina. Then, an initial attempt to reposition the uterus abdominally by applying tension on both round ligaments (*Huntington* procedure) failed. Afterward, a linear incision was made on the constriction ring and advanced on the posterior wall of the uterus while applying gentle upward pressure vaginally (*Haultain* procedure). The uterus was subsequently reinverted to its normal anatomy by placing a finger abdominally through the myometrial incision and exerting pressure on the fundus (Figure 3). Finally, a total abdominal hysterectomy with bilateral salpingo-oophorectomy was performed. The estimated blood loss was 400 ml.

**Figure 4:**
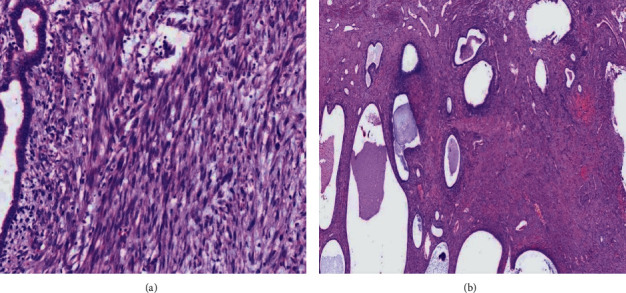
Histological section reveals (a) interlacing fascicles of bland spindle cells with adjacent glandular structures suggestive of a leiomyoma with focuses of adenomyosis and (b) cystically dilated glands of different sizes lined by bland columnar cells in muscular stroma suggestive of adenomyosis.

The hemoglobin level determined 6 hours after the surgery was found to be 7.0 g/dl, and the patient was transfused with 3 units of blood. She recovered well and was discharged on postoperative day 7. Histopathological examination of the specimen showed leiomyoma with adenomyosis. There was no evidence of malignancy (Figure 4). The patient had follow-up visits at 2 and 6 weeks. She has no constipation, urinary frequency, urgency, retention, or involuntary loss. An abdominal exam showed a clean and healed surgical incision. On pelvic examination, the vaginal cuff appeared healthy and well suspended at the level of the ischial spines. There was no anterior or posterior vaginal wall defect. The patient was reassured and was linked to the diabetic clinic to continue her follow-up.

## 3. Discussion

Nonpuerperal uterine inversion is a rare condition. A review of published literature from 1940 to 2017 reported 170 cases of nonpuerperal uterine inversions [4]. In general, nonpuerperal uterine inversion present after 45 years and it is usually associated with the presence of a polypoid uterine mass [2].

Nonpuerperal uterine inversion can easily be misdiagnosed, and in most instances, the inversion may not be noticed until the time of surgery. There must be a strong clinical suspicion to diagnose this condition. History of protruding vaginal mass and bleeding are usually the presenting symptoms. The size of the tumor and abnormally high intra-abdominal pressure (due to coughing, sneezing, constipation, etc.) may promote uterine inversion. A clinical examination might help in the diagnosis when a protruding vaginal mass is accompanying the absence of the uterus in its normal position or palpation of fundal depression on bimanual examination [2, 5]. Our case presented with a complaint of bleeding and large protruding vaginal mass immediately preceded by urinary retention and intense straining.

Imaging studies can facilitate the diagnosis of nonpuerperal uterine inversion. Ultrasound represents the first-line imaging modality. Indentation of the uterine fundus and a depressed longitudinal groove extending from the fundus to the center of the inverted uterus are ultrasonographic findings consistent with uterine inversion [6].

The 3D power Doppler has been used more recently in the diagnosis of uterine inversion as it can clearly show the changes in the uterine artery course in relation to the uterus. A U-turn sign, showing a central course of the main uterine vessels instead of their normal anatomical peripheral location laterally alongside the corpus of the uterus, may represent a novel and pathognomonic sign of uterine inversion [7].

MRI is the best imaging modality to diagnose uterine inversion and can show the anatomic anomalies more accurately than CT. MRI is useful not only in diagnosis but also to characterize the uterine mass [5]. On MRI scans, “U”-shaped uterine cavity, thickened and inverted uterine fundus are indicative of uterine inversion [8]. Identifying the round ligaments and fallopian tubes protruding centrally from the top of the uterus is also highly suggestive of inversion [9]. However, MRI may not be readily available in most hospital settings in developing countries. In our case, the diagnosis was based on clinical findings of protruding vaginal mass, inability to see the cervical os, and no uterine echo on pelvic ultrasonography. We deferred other imaging procedures as the patient was having continuous bleeding and severe pain.

Treatment of nonpuerperal uterine inversion depends on the patient's desire for future fertility and the nature of the intrauterine prolapsed mass (benign or malignant). If future fertility is desired and malignancy is excluded, surgical procedures to reposition and preserve the uterus should be attempted [5, 10]. Thus, there is a need for histologic diagnosis prior to definitive surgery. This is particularly important as new evidence suggests a higher association of nonpuerperal uterine inversion with malignancy than initially thought [2]. A recent review shows that about 30% of nonpuerperal uterine inversions were due to malignancy [4]. In our case, due to the age of the patient and the characteristics of the mass, the possibility of malignancy was entertained. Furthermore, childbearing was no longer pertinent to the patient. Thus, she was counseled on the options of treatment and hysterectomy with bilateral salpingo-oophorectomy was considered more appropriate.

Several surgical techniques have been described. Two abdominal (*Huntington* and *Haultain*) and two vaginal (*Spinelli* and *Kustner*) techniques have long been used to reinvert the uterus [3]. More recently, laparoscopic and robotic surgeries are emerging as novel options [11–13]. In our case, we used a combined vaginal and abdominal approach. The protruding mass was removed vaginally. This facilitated repositioning of the uterus abdominally using the *Haultain* technique assisted with a gentle and sustained push from below. Once repositioning was successful, hysterectomy with bilateral salpingo-oophorectomy was carried out using standard procedural steps. Recent reviews also show that a combined vaginal and abdominal approach, to first remove the cause of inversion vaginally and then proceed to uterine repositioning abdominally, is an effective surgical strategy [5]. The repositioning of the uterus to the exact anatomy is vital before proceeding to hysterectomy. It is assumed that hysterectomy would be technically easier on a normally positioned uterus as repositioning would restore normal anatomy most surgeons are familiar with [10, 14].

## 4. Conclusion

This case highlights that nonpuerperal uterine inversion should be included in the differential diagnosis when a patient presents with protruding vaginal mass and bleeding. Carefully conducted clinical examination including examination under anesthesia and pelvic ultrasonography can be valuable tools to reach at a diagnosis. Clinicians need to familiarize themselves with the principles of recommended surgical techniques in order to select the best approach. A combined vaginal and abdominal surgical approach can facilitate repositioning and/or hysterectomy when there is a large protruding vaginal mass.

## Data Availability

The data supporting the conclusions of this report are included within the manuscript.

## References

[1] Abouleish E., Ali V., Joumaa B., Lopez M., Gupta D. (1995). Anaesthetic management of acute puerperal uterine inversion. *British Journal of Anaesthesia*.

[2] Gomez-Lobo V., Burch W., Khanna P. C. (2007). Nonpuerperal uterine inversion associated with an immature teratoma of the uterus in an adolescent. *Obstetrics and Gynecology*.

[3] Bayer-Zwirello L. A., O'Grady J. P., Gimovsky M. L. (2008). The third stage. *Operative Obstetrics*.

[4] Rosa Silva B., de Oliveira Meller F., Uggioni M. L. (2018). Non-puerperal uterine inversion: a systematic review. *Gynecologic and Obstetric Investigation*.

[5] Martin A., Tranoulis A., Sayasneh A. (2020). Uterine inversion secondary to a large prolapsed leiomyoma: diagnostic and management challenges. *Cureus*.

[6] Krissi H., Peled Y., Efrat Z., Goldshmit C. (2011). Ultrasound diagnosis and comprehensive surgical treatment of complete non-puerperal uterine inversion. *Archives of Gynecology and Obstetrics*.

[7] Zohav E., Anteby E. Y., Grin L. (2020). U-turn of uterine arteries: a novel sign pathognomonic of uterine inversion. *Journal of Ultrasound*.

[8] Lewin J. S., Bryan P. J. (1989). MR imaging of uterine inversion. *Journal of Computer Assisted Tomography*.

[9] Moulding F., Hawnaur J. M. (2004). MRI of non-puerperal uterine inversion due to endometrial carcinoma. *Clinical Radiology*.

[10] Herath R. P., Patabendige M., Rashid M., Wijesinghe P. S. (2020). Nonpuerperal uterine inversion: what the gynaecologists need to know?. *Obstetrics and Gynecology International*.

[11] Auber M., Darwish B., Lefebure A., Ness J., Roman H. (2011). Management of nonpuerperal uterine inversion using a combined laparoscopic and vaginal approach. *American Journal of Obstetrics and Gynecology*.

[12] Zhang X., Sun L., Chen X., Hua K. (2015). Uterus preserving reposition of non-puerperal uterine inversion under laparoscope: a case report and literature review. *Gynecologic and Obstetric Investigation*.

[13] Zechmeister J. R., Levey K. A. (2011). Successful robotically assisted laparoscopic correction of chronic uterine inversion. *Journal of Minimally Invasive Gynecology*.

[14] Della Corte L., Giampaolino P., Fabozzi A., di Spiezio Sardo A., Bifulco G. (2019). An exceptional uterine inversion in a virgo patient affected by submucosal leiomyoma: case report and review of the literature. *The Journal of Obstetrics and Gynaecology Research*.

